# Proliferation-Attenuating and Apoptosis-Inducing Effects of Tryptanthrin on Human Chronic Myeloid Leukemia K562 Cell Line *in Vitro*

**DOI:** 10.3390/ijms12063831

**Published:** 2011-06-10

**Authors:** Shan Miao, Xiaopeng Shi, Hai Zhang, Siwang Wang, Jiyuan Sun, Wei Hua, Qing Miao, Yong Zhao, Caiqin Zhang

**Affiliations:** 1 Institute of Materia Medica, School of Pharmacy, Fourth Military Medical University, #17 West Changle Road, Xi’an 710032, China; E-Mails: miaoshan@fmmu.edu.cn (S.M.); sunjiy@fmmu.edu.cn (J.S.); miaoqing@fmmu.edu.cn (Q.M.); 2 Department of Pharmacy of Xijing Hospital, Xi’an 710032, China; E-Mail: shixiaop@fmmu.edu.cn; 3 Laboratory Animal Research Center, Xi’an 710032, China; E-Mails: hzhang@fmmu.edu.cn (H.Z.); zhaoy_mail@yahoo.com.cn (Y.Z.); zhangcaiqin-beibei@163.com (C.Z.); 4 Department of Obstetrics Gynecology, Xijing Hospital, Xi’an 710032, China; E-Mail: huawei@fmmu.edu.cn

**Keywords:** tryptanthrin, chronic myeloid leukemia, proliferation, apoptosis, K562 cells

## Abstract

Tryptanthrin, a kind of indole quinazoline alkaloid, has been shown to exhibit anti-microbial, anti-inflammation and anti-tumor effects both *in vivo* and *in vitro*. However, its biological activity on human chronic myeloid leukemia cell line K562 is not fully understood. In the present study, we investigated the proliferation-attenuating and apoptosis-inducing effects of tryptanthrin on leukemia K562 cells *in vitro* and explored the underlying mechanisms. The results showed that tryptanthrin could significantly inhibit K562 cells proliferation in a time- and dose-dependent manner as evidenced by MTT assay and flow cytometry analysis. We also observed pyknosis, chromatin margination and the formation of apoptotic bodies in the presence of tryptanthrin under the electron microscope. Nuclei fragmentation and condensation by Hoechst 33258 staining were detected as well. The amount of apoptotic cells significantly increased whereas the mitochondrial membrane potential decreased dramatically after tryptanthrin exposure. K562 cells in the tryptanthrin treated group exhibited an increase in cytosol cyt-c, Bax and activated caspase-3 expression while a decrease in Bcl-2, mito cyt-c and pro-caspase-3 contents. However, the changes of pro-caspase-3 and activated caspase-3 could be abolished by a pan-caspase inhibitor ZVAD-FMK. These results suggest that tryptanthrin has proliferation-attenuating and apoptosis-inducing effects on K562 cells. The underlying mechanism is probably attributed to the reduction in mitochondria membrane potential, the release of mito cyt-c and pro-caspase-3 activation.

## 1. Introduction

Chronic myeloid leukemia (CML) is initiated by the aberrant pluripotent hematopoietic stem cell (HSC) growth and apoptosis characterized by accumulation of immature granulocytes in the peripheral blood and bone marrow. The highly proliferative feature of leukemia cells distinguishes them from most normal cells. Therefore, inhibiting HSC proliferation might promote leukemia cell apoptosis and cell cycle arrest, which is clinically beneficial in the therapeutic strategy of CML [1].

The disturbance of balance between HSC proliferation and apoptosis plays a critical role in the pathogenesis of leukemia and apoptosis-inducing mechanism and is clinically used to treat leukemia [2,3]. Imatinib mesylate, an orally administrated tyrosine kinase inhibitor is recommended as the first-line drug for bcr-abl fusion gene positive CML patients. Imatinib mesylate competitively inhibits ATP binding to bcr-abl tyrosine kinase and promotes CML cells apoptosis. Apoptosis, known as programmed cell death (PCD), is essential for normal development and cell homeostasis and it participates in many pathological processes [4]. It is morphologically characterized by chromatin margination and the formation of apoptotic bodies [5]. Recently, mitochondria depolarization has been recognized as an early event in the process of apoptosis [6,7]. Decreased mitochondria membrane potential inhibits ATP generation, results in oxidative stress and promotes mitochondria dysfunction. Mitochondrial cyt-c (mito cyt-c) leakage into the cytoplasm would activate pro-caspase and caspase cascade, especially the caspase-3, which facilitates cell apoptosis in return [8].

Although a number of chemotherapy regimens can induce leukemia cell apoptosis and increase the overall survival time, the recurrence of leukemia due to micro-residential disease (MRD) remains a common problem. Meanwhile, the clinically used chemotherapeutics (such as cyclophosphamide, CTX) have severe and inevitable adverse effects. Therefore, it is important and urgent to look for an effective treatment for MRD and the naturally synthetic compounds seems to offer more clinical benefits in the treatment and prevention of leukemia [9]. Tryptanthrin is a naturally occurring substance firstly isolated by Honda *et al.* from the indigo plant *Polygonum tinctorium*, and they reported that tryptanthrin has a broad spectrum of biological functions, such as anti-inflammatory, anti-fungal and anti-bacterial effects [10–12]. Increasing evidence showed tryptanthrin has a wide range of downstream targets that regulate tumor-associated cell processes including cell growth, cell cycle progression and survival. Previous studies have demonstrated that tryptanthrin exhibited anti-tumor effects on various kinds of solid tumors and it is suggested that tryptanthrin inhibits multiple drug resistance to chemotherapies and reverses doxorubicin resistance during breast cancer therapy [13]. Meanwhile, tryptanthrin is non-toxic and free of adverse effects on various animal models using different models of administration [10,12]. Thus it provides many clinical benefits during cancer therapy. However, its therapeutic effect on human CML cells has not been fully understood and the underlying mechanisms are beyond dispute.

In the present study, we explored whether tryptanthrin has proliferative-attenuating and apoptosis-inducing effects on human CML cells. We show that tryptanthrin inhibits mitochondria membrane potential in the human CML cell line K562, accompanied by cell cycle arrest, caspases activation, apoptosis induction and proliferation inhibition.

## 2. Materials and Methods

### 2.1. Reagents

**Figure 1:**
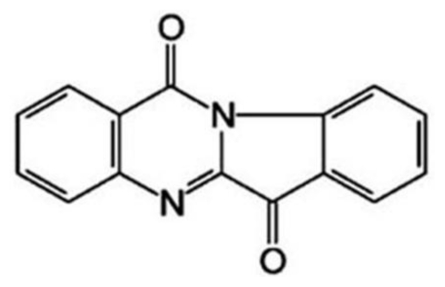
Chemical structure of tryptanthrin.

*Medicine*: tryptanthrin (Batch No. 20061211; Figure 1), 6,12-dihydro-6,12-dioxoindolo-(2,1-b)- quinazoline, was provided by College of Life Science of Northwest University (Shaanxi province, China), with purity over 99.7%. Tryptanthrin was dissolved in dimethyl sulfoxide (DMSO) and diluted with culture medium.

*Materials*: RPMI medium and fetal bovine serums (FBS) were purchased from Gibco. MTT [3-(4,5-dimethylthiazol-2-yl)-2,5-diphenyl tetrazolium bromide], Hoechst 33258 and Rhodamine 123 fluorescent dye were from Sigma. Annexin-V-FITC and pyrimidine of iodinate (PI) were from Bio Vision Inc. RIPA Lysis buffer was from Runde Biotech Co. Mouse anti-human β-actin monoclonal IgG was from Sigma. Rabbit anti-human Bcl-2, Bax, cyt-c and caspase-3 monoclonal antibodies and the respective HRP-labeled secondary antibodies were products from Santa. ZVAD-FMK was purchased from Biomol International LP. CTX was obtained from the Institute of Pharmacy, Xijing Hospital, Fourth Military Medical University.

### 2.2. Cell Culture and Treatment

K562 human CML cells (*bcr*-*abl* fusion gene positive) were provided by Laboratory Animal Research Center of the Fourth Military Medical University (Shaanxi province, China). Cells were cultured in RPMI 1640 medium supplemented with 10% FBS, penicillin (100 U/mL) and streptomycin (100 μg/mL) in an atmosphere with 5% CO_2_ at 37 °C. In all experiments, exponentially growing cells were used.

### 2.3. MTT Assay

Cell proliferation was assessed using the MTT assay as previously described. Briefly, 5 × 10^3^ cells were incubated in 96-well plates in the presence of 0, 0.39, 0.78, 1.56, 3.12, 6.25, 12.5 and 25 μg/mL tryptanthrin for 24 h and 48 h in a final volume of 200 μL. At the end of the treatment, 20 μL MTT (5 mg/mL dissolved in PBS) was added to each well and incubated for an additional 4 h at 37 °C. The purple-blue MTT formazan precipitate was dissolved in 100 μL of DMSO. The activity of the mitochondria, reflecting cellular growth and viability, was evaluated by measuring the optical density at 570 nm. The cell survival rate was calculated as A_treatment group_/A_control group_ × 100%.

### 2.4. Hoechst 33258 Fluorescent Staining

K562 cells from exponentially growing cultures were seeded in 24-well culture plates. The cells received 0 (control), 6.25, 12.5 and 25 μg/mL tryptanthrin or vehicle (0.5% DMSO) for 48 h. To verify the apoptosis-inducing effect of tryptanthrin, CTX (0.5 μg/mL) was selected as a positive control. K562 cells were incubated with CTX for 48 h as well. The cells were then washed in ice-cold phosphate-buffered saline (PBS), and fixed in a solution of methanol-acetic acid (3:1, v/v) for 15 min at 4 °C. To identify the apoptotic K562 cells, they were stained with Hoechst 33258 (5 μg/mL in PBS) for 5 min at room temperature. The nuclei structure of the cells was examined by Olympus fluorescence microscopy with an excitation wavelength of 340 nm and an emission wavelength of 460 nm. Five fields were randomly selected and the apoptotic cells were observed at 200× magnification.

### 2.5. Transmission Electron Microscopy

K562 cells were incubated with tryptanthrin and CTX under the same conditions as previously described. The cells were collected and cell pellets were fixed with 2% glutaraldehyde in 0.1% sodium cacodylate buffer, pH 7.4 for 12 h at 4 °C. Fixation was followed by 3–5 min washes with 0.1% sodium cacodylate buffer, pH 7.4. Cells were post-fixed with a solution containing 1% osmium tetroxide and 2% K_4_Fe, stained with 1% uranyl acetate, and pelleted in 2% agar. Pellets were dehydrated in graded ethanol solution and embedded in spur resin. Ultra thin (60 nm) sections were cut on a Reichert Ultra cut microtome, collected on Rhodanimu 400-mesh grids, post-stained with uranyi acetate and lead citrate, and washed with water. The sections were examined in transmission election microscope (JEM-2000EX).

### 2.6. Annexin-V/PI Staining, Apoptosis and Cell Cycle Determination by Flow Cytometry

Cells in each group were collected and diluted to the concentration of 1.0 × 10^6^/mL. The cells were washed twice and suspended in 200 μL PBS. After that, cells were incubated with 10 μL Annexin-V-FITC and 5 μL PI for 30 min at 4 °C. The cells undergoing apoptosis were detected by FCM (Beckman Coulter, USA). For the detection of cell cycle, cells were incubated with the solution containing RNase and PI for 30 min. At least 10^4^ cells were analyzed for each determination. The percentages of cells in G_0_/G_1_, S and G_2_/M cell cycle phases were calculated by the Modfit 3.0 program (Verity Software House).

### 2.7. Measurement of Mitochondrial Membrane Potential (Δψm)

Since the evidence that cells undergoing apoptosis exhibit reduced mitochondrial membrane potential (MMP), MMPs were measured to reflect the amount of apoptotic cells. In brief, cells were collected and washed twice in PBS and stained with the fluorochrome Rhodamine 123 (5 mg/L) for 1 h at 37 °C. After that, cells were centrifuged and washed twice in ice-cold PBS, resuspended in PBS and analyzed by FCM.

### 2.8. Assay of Bcl-2, Bax, Cyt-c, Procaspase-3 and Caspase-3

After exposed to 6.25, 12.5 and 25 μg/mL tryptanthrin for 48 h, respectively, cells were collected and washed in ice-cold PBS, and lysed in RIPA buffer (50 mM Tris-HCl pH 7.4, 150 mM NaCl, 1% NP-40, 0.1% SDS, 1 mM PMSF) for extraction of total protein. We used immunoblotting to demonstrate cleavage of pro-caspase-3 to its catalytically active subunits (p20 and p17) as induced by mitochondrial cyt-c leakage. To investigate whether tryptanthrin-induced K562 cells apoptosis was caspase-dependent, ZVAD-FMK (a pan-caspase inhibitor) at the concentration of 20 μmol/L was added into the culture medium before tryptanthrin exposure in another group. After centrifugation for 10 min at 12,000 g, the supernatants were collected and the total proteins were quantified with the Bradford assay Kit (Beyotime biotechnology, China). Equal amount of protein was separated by SDS-PAGE and transferred to nitrocellulose membranes at 400 mA for 1 h. Membranes were stained with 0.5% Ponceau in 1% acetic acid for confirmation. Blots were blocked for 2 h in TBST (10 mM Tris-HCl, pH 7.4, 150 Mm NaCl, 0.05% Tween-20) containing 5% fat-free dried milk and then incubated with the primary antibodies (Rabit anti-Bcl-2, Bax, caspase-3 IgG, respectively) for 12 h and then incubated with respective HRP-labeled secondary antibodies. Signals were detected using the enhanced chemiluminescence system (Minipore).

To detect cyt-c in mitochondrial and cytosolic fractions during tryptanthrin treatment, mitochondria was isolated as previously described [14]. Briefly, K562 cells were treated as above and converted to spheroplasts by zymolyase treatments (ICN). Spheroplasts were disrupted by an osmotic shock and a hand-potter homogenization to preserve the outer mitochondrial membrane, and the mitochondrial fraction was recovered after a series of differential centrifugation. The supernatant was collected and removed to determine cyt-c in cytoplasma. Where indicated, mitochondria were incubated in the presence of 0.1% Triton X-100 for 15 min at 4 °C and centrifuged for 15 min at 10,500 g and the pellet was treated by SDS-PAGE and Western blot analysis.

### 2.9. Statistical Analysis

Clampfit software and ORIGIN6.1 software were used to analyze the data. Values are expressed as mean ± SD. One-way analysis of variance was employed to determine the statistical significance of the different groups. Significance level was set at *P* < 0.05.

## 3. Results

### 3.1. The Growth Suppression Effect of Tryptanthrin on K562 Cells

**Figure 2:**
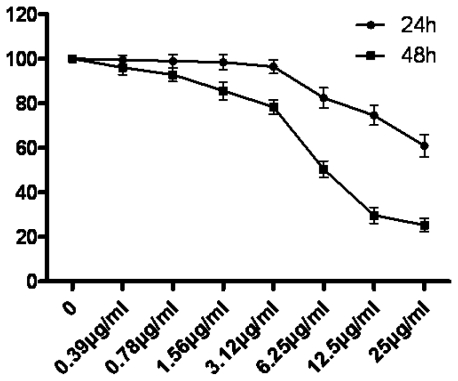
Effect of tryptanthrin on the survival rate of K562 cells. Cells were treated with various concentrations of tryptanthrin for 24 h and 48 h. The cell survival rate was determined by MTT assay. Each data point is the mean of three replicates; bars represent the standard deviation.

As shown in Figure 2, we observed that tryptanthrin suppressed K562 cell growth using MTT staining. The cell survival rates decreased in parallel with elevating tryptanthrin concentrations. Meanwhile, the cell survival rate in the 48 h group was lower than that in the 24 h group under the same tryptanthrin concentration. Tryptanthrin exhibited a growth suppression effect on K562 cells in a time- and dose-dependent manner with a 50% decrease in cell proliferation at the concentration (IC_50_) of 8.8 μg/mL after 48 h treatment.

### 3.2. Determination of Apoptotic K562 Cells by Hoechst 33258 Staining

**Figure 3:**
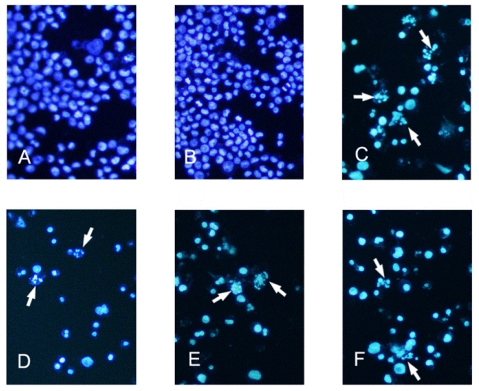
Fluorescent staining of nuclei in tryptanthrin-treated K562 cells by Hoechst 33258. Cells were incubated in the medium without tryptanthrin (**A**) or with 0.5% DMSO (**B**), CTX (**C**) and with 6.25, 12.5, 25 μg/mL tryptanthrin (**D**, **E** and **F**) for 48 h, respectively. Fragmented or condensed nuclei could be observed at 200× magnification in the tryptanthrin-treated group as indicated by the arrows.

Stained with Hoechst 33258, the K562 cells in the control and 0.5% DMSO groups exhibited small and round normal nuclei (Figure 3A,B). In contrast, cells treated with CTX showed signs of apoptosis, including highly condensed and fragmented nuclei (Figure 3C). Meanwhile, cells incubated with tryptanthrin at the concentration of 6.25, 12.5 and 25 μg/mL for 48 h also exhibited similar apoptotic signs (Figure 3C–F). The amount of cells undergoing apoptosis in each randomly selected field increased with the content of tryptanthrin and CTX induced more apoptotic cells than 25 μg/mL tryptanthrin.

### 3.3. K562 Cells Ultra Structure

**Figure 4:**
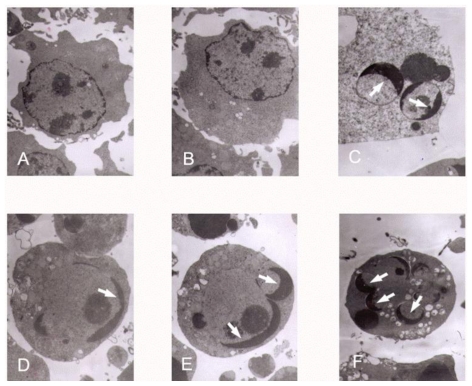
Transmission electron micrographs of K562 cells treated with different concentrations of tryptanthrin. No abnormal changes were observed in the control (**A**) and 0.5% DMSO (**B**) groups. Pyknosis, chromatin margination and the formation of apoptotic bodies (white arrows) were clearly observed in the presence of CTX (**C**) and tryptanthrin at the concentrations of 6.25 (**D**), 12.5 (**E**) and 25 (**F**) μg/mL, respectively.

We observed cell swelling, rarefaction and membrane damage in K562 cells after tryptanthrin and CTX treatment. Dilation of the endoplasmic reticulum was also observed (not shown in Figure 4). Compared with the control and 0.5% DMSO group, nuclei in the tryptanthrin and CTX treated group manifested apoptotic changes, including pyknosis, chromatin margination and the formation of apoptotic bodies (Figure 4C–F). No apoptotic cells were observed in the control and 0.5% DMSO groups (Figure 4A,B).

### 3.4. Tryptanthrin-Induced K562 Cell Apoptosis and FCM Analysis

**Figure 5:**
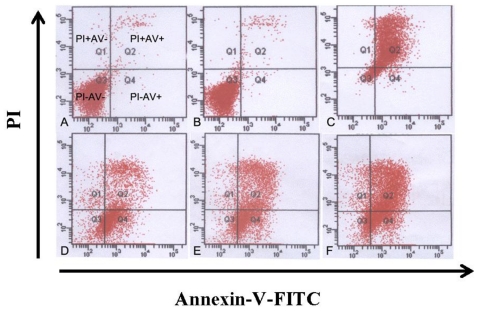
Tryptanthrin caused strong K562 cells apoptosis. Representative FCM analysis scattergrams of Annexin V/PI stained control, 0.5% DMSO and treatment with CTX and 6.25, 12.5 and 25 μg/mL tryptanthrin for 48 h cells showed four different cell populations marked as: live cell population (PI−AV−), early apoptosis (PI−AV+), late apoptosis (PI+AV+) and dead cells (PI+AV−). Cells treated with no tryptanthrin (**A**), 0.5% DMSO (**B**), CTX (**C**) and 6.25, 12.5 and 25μg/mL tryptanthrin, respectively (**D**, **E** and **F**).

K562 cells cultured with trytanthrin were analyzed for apoptosis by Annexin V and PI (Annexin V/PI) dual staining. Annexin V/PI staining in the control group showed a large viable cell population (marked as PI−AV−) and a small amount of early apoptotic (PI−AV+), late apoptotic (PI+AV+), and dead cells (PI+AV−). Tryptanthrin at the concentrations of 6.25, 12.5 and 25 μg/mL resulted in a strong shift from viable cells to early and late apoptotic cell population with little change in the dead cell population. FCM analysis showed tryptanthrin induced K562 cells apoptosis in a dose-dependent manner. About 21.9% ± 2.3%, 33.4% ± 3.6% and 52.2% ± 4.7% PI+AV+ cells were detected in the 6.25, 12.5 and 25 μg/mL tryptanthrin groups while that proportions were 0.2% ± 0.01%, 0.6% ± 0.01% and 73.0% ± 6.7% in the control group, 0.5% DMSO and CTX group, respectively. There was a significance difference in the amount of apoptotic cells in the tryptanthrin-treated groups compared with the control group. (Figure 5, data not shown).

### 3.5. Changes in Cell Cycle Progression

**Figure 6:**
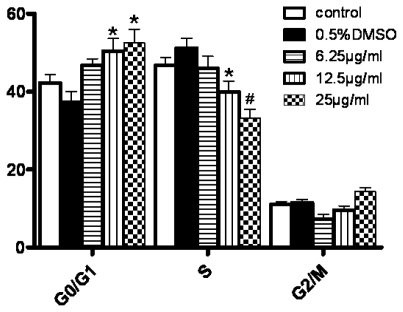
Cell cycle distribution analysis after tryptanthrin exposure. The proportions of G_0_/G_1_, S and G_2_/M phase cells were measured by Modfit 3.0 program. * *P* < 0.05, ^#^ *P* < 0.01.

To further understand the mechanisms of tryptanthrin-induced K562 cell apoptosis, we analyzed the cell cycle phase distribution. Cells were stained with Annexin-V-FITC and PI and analyzed after 48 h treatment with tryptanthrin. As shown in Figure 6, tryptanthrin arrested K562 cells in G_0_/G_1_ phase. The proportion of S phase cells in the control and 0.5% DMSO groups was relatively high, suggesting the K562 cells were highly proliferative. After tryptanthrin treatment, the proportion of S phase cells in each tryptanthrin-treated group was markedly decreased compared with the control group. We also found the proliferation-inhibiting effect of tryptanthrin was maximal at the dose of 25 μg/mL (S phase proportion 33.2% ± 2.3% *vs.* 46.7% ± 2.0% in the control group, *P* < 0.01). Compared with the control group, the cell populations of tryptanthrin-treated groups in the G_0_/G_1_ phase significantly increased to 52.5% ± 3.6% (*P* < 0.05). These results suggested that the breakdown of DNA synthesis resulted in cell killing and the breakdown was caused due to cell cycle arrest. There was no significant change in the G_2_/M phase after tryptanthrin exposure.

### 3.6. Changes in Mitochondrial Membrane Potentials

**Figure 7:**
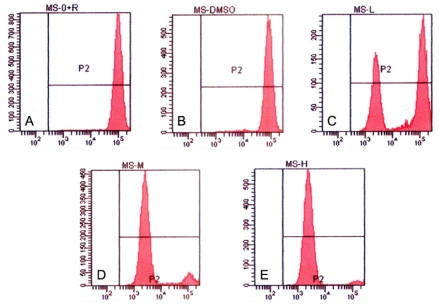
Induction of K562 cell mitochondrial membrane collapse in the presence of tryptanthrin. Mitochondrial membrane potential was assessed by FCM after Rhodamine 123 staining. Rhodamine 123 enriched mainly in mitochondria showing red fluorescence reflecting changes of mitochondrial membrane potential. Cells were treated with no tryptanthrin (**A**), 0.5% DMSO (**B**) and 6.25, 12.5 and 25μg/mL tryptanthrin, respectively (**C**, **D** and **E**).

Tryptanthrin treated cells were examined for fluorescence density after Rhodamine 123 staining. The drop in the red fluorescence, suggesting the disruption of MMP, was detected by FCM. The MMPs in each tryptanthrin-treated group were significantly lower than in the control and 0.5% DMSO groups. And the reduction of MMP in the tryptanthrin-treated groups was in parallel with the tryptanthrin concentrations (Figure 7).

### 3.7. Expression of Bcl-2, Bax, Cyt-c and Caspase-3

**Figure 8:**
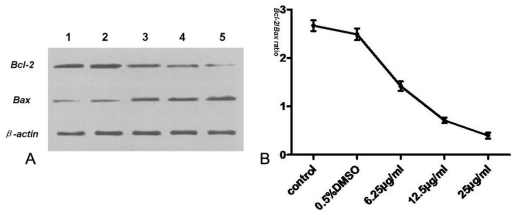
Western blot assay of *Bcl-2*, *Bax* and *β-actin* protein expression in K562 cells. (**A**) expression of *Bcl-2* and *Bax* after tryptanthrin exposure. *β-actin* was used for normalization and verification of protein loading; (**B**) the *Bcl-2*/*Bax* ratio in the control, 0.5% DMSO and each tryptanthrin-treated groups. Lane 1: control; lane 2: 0.5% DMSO; lane 3: 6.25 μg/mL tryptanthrin; lane 4: 12.5 μg/mL tryptanthrin; lane 5: 25 μg/mL tryptanthrin.

To further provide insight into tryptanthrin-induced cell apoptosis, we determined the Bcl-2, Bax, cytosol and mitochondrial cyt-c, pro-caspase-3 and activated caspase-3 expression 48 h after tryptanthrin exposure. As shown in Figure 8, Bcl-2 protein was highly expressed while Bax protein was relatively minimally detected in the control and 0.5% DMSO groups. The Bcl-2/Bax ratio was relatively high in the two groups. However, the content of Bcl-2 decreased whereas Bax increased significantly after tryptanthrin exposure. The Bcl-2/Bax ratio was reversed in each tryptanthrin-treated group.

**Figure 9:**
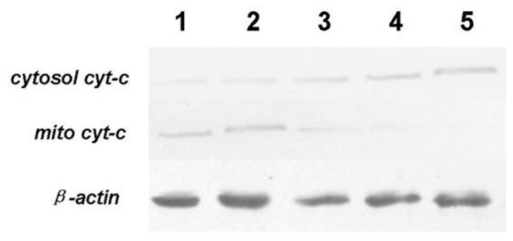
Western blot assay of *cytosol cyt-c* and *mito cyt-c* in K562 cells. *β-actin* was used as an internal control. The expressions of *cytosol cyt-c* were very low in the control and 0.5% DMSO group. Tryptanthrin administration could lead to mito *cyt-c* leakage into the cytoplasm and increase *cytosol* contents in a dose dependent manner. Lane 1: control; lane 2: 0.5%DMSO; lane 3: 6.25 μg/mL tryptanthrin; lane 4: 12.5 μg/mL tryptanthrin; lane 5: 25 μg/mL tryptanthrin.

Cyt-c can activate the pro-caspases when released from mitochondria into the cytoplasm and it is responsible for apoptosis through interaction with protease-activating factors. Normally, the permeability of the mitochondrial membrane was quite low. Cyt-c was confined in the mitochondria and defended from leaking into cytosolic fractions. Therefore, caspases existed in the form of pro-caspases in normal cells. As shown in Figure 9, the expressions of cytosol cyt-c in the control and 0.5% DMSO groups were very low, whereas mito cyt-c was relatively high. However, as demonstrated above, tryptanthrin could reduce the MMPs and increase the mitochondrial membrane permeability. Therefore, tryptanthrin led to increased mito cyt-c leakage and elevate cytosol cyt-c contents.

**Figure 10:**
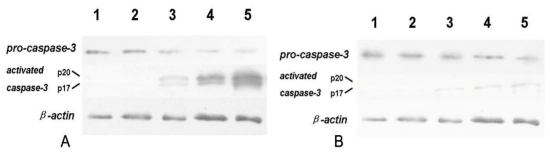
Western blot assay of *pro-caspase-3* and *activated caspase-3* in K562 cells. *β-actin* was used as an internal control. *Caspase-3* exists as its inactive form *pro-caspase-3* and there are no detectable active *caspase-3* subunits in K562 cells. Administration of tryptanthrin induces mito *cyt-c* leakage and activates *pro-caspase-3*. The expression of active *caspase-3* subunits p20 and p17 were remarkably elevated while *pro-caspase-3* contents decreased after tryptanthrin exposure. However, addition of a pan-caspase inhibitor ZVAD-FMK significantly attenuated the *pro-caspase-3* activation in response to tryptanthrin. The contents of p20 and p17 remarkably decreased in the presence of ZVAD-FMK. (**A**) Lane 1: control; lane 2: 0.5%DMSO; lane 3: 6.25 μg/mL tryptanthrin; lane 4: 12.5 μg/mL tryptanthrin; lane 5: 25 μg/mL tryptanthrin; (**B**) Lane 1: control; lane 2: ZVAD-FMK; lane 3: ZVAD-FMK + 6.25 μg/mL tryptanthrin; lane 4: ZVAD-FMK + 12.5 μg/mL tryptanthrin; lane 5: ZVAD-FMK + 25 μg/mL tryptanthrin.

Caspase activation is initiated by cytosol cyt-c. As shown in Figure 10A, tryptanthrin was capable of inducing activation and cleavage of pro-caspase-3 *in vitro*, upon elevated mito cyt-c leakage. We observed the content of pro-caspase-3 significantly decreased after tryptanthrin administration. We then evaluated the expression of activated caspase-3 subunits p20 and p17. Immunoblotting of activated caspase-3 using a specific monoantibody against caspase-3 revealed no detectable p20 and p17 found in the control and 0.5% DMSO groups. Tryptanthrin administration significantly increased the content of p20 and p17 in a dose-dependent manner. As illustrated in Figure 10B, addition of 20 μmol/L ZVAD-FMK (a pan-caspase inhibitor) significantly inhibited the activation of pro-caspase-3 in response to tryptanthrin exposure.

## 4. Discussion

Cancer chemoprevention is defined as inhibition of tumor initiation, promotion and progression by employing pharmacologic or natural agents that prevent the metabolic activation of procarcinogens. A number of natural phytochemicals are shown to have anti-cancer properties [15]. Their mechanisms responsible for executing the anti-proliferative effects mainly include blockade of tumor cell expansion and induction of tumor cell differentiation or apoptosis. K562 cells are derived from human CML and express the bcr/abl kinase. bcr/abl kinase enables the CML cells to growth in a uncontrolled way by inducing p38 MAPK, Ras, Jun, PI-3K/Akt and STAT5 pathways, and blockage of bcr/abl kinase by imatinib mesylate has been recognized as the target therapy for CML [2,16–18]. Gangemi *et al.* showed that bcr/abl exerted its anti-apoptotic effect against diverse apoptotic stimuli through blockage of mitochondrial release of cyt-c and activation of pro-caspase-3, the molecular bases of the delayed response of K562 cells resided downstream of Bcl-2 phosphorylation [19,20].

Tryptanthrin is extracted from medical herbs *Polygonum tinctorium* and a number of studies have demonstrated the application of the herbal extracts exert abundant benefits in the management of many disorders [6,21]. As the bioactive ingredient of the medical herbs, tryptanthrin has been reported to show various biological activities, including anti-microorganisms and anti-inflammation [10,11,22]. Recently, it was found tryptanthrin suppresses growth and proliferation of transformed or malignant cells through induction of apoptosis on various kinds of solid tumors by inhibiting NO synthases (NOS) and cyclooxygenase (COX) activity [10]. Yu *et al.* reported tryptanthrin alleviates multiple drugs resistance (MDR) and improves doxorubicin sensitivity in breast cancer cells [13]. However, whether tryptanthrin has similar effects on CML and the underlying mechanisms remained unclear. The primary aim of the present study was to investigate the proliferation-attenuating and apoptosis-inducing effects of tryptanthrin on CML K562 cells through screening the best inducer and then proving its machinery.

In our experiments, we showed that tryptanthrin produced a growth suppression effect on K562 cells in a time- and dose-dependent manner. Trytpanthrin led to 50% decrease in K562 cell proliferation at the dosage of 8.8 μg/mL after 48 h treatment. The K562 cells undergoing apoptosis are evidenced by morphological changes observed under fluorescent microscopy and transmission election microscopy. Nuclear fragmentation and chromosome margination were clearly observed in the CTX and tryptanthrin-treated groups. Consistent with the observation, our flow cytometry analysis showed tryptanthrin at the concentrations of 6.25, 12.5 and 25μg/mL caused 21.9% ± 2.3%, 33.4% ± 3.9% and 52.2% ± 4.7% cells in the late apoptotic zone bound (marked as PI+AV+) respectively. These results indicate that tryptanthrin exerts apoptosis-inducing effect as CTX does. Cell cycle distribution analysis showed that tryptanthrin inhibited proliferation via blocking cell cycle progression at the G_0_/G_1_ phase and subsequently progressing into apoptosis. There was a significant increase in G_0_/G_1_ phase proportion and a remarkable decrease in S phase proportion in the presence of 12.5 and 25 μg/mL tryptanthrin compared with the control group.

Tryptanthrin regulates cell survival and apoptosis through a phosphorylation cascade that primarily alters the function of transcription factors that regulate pro-apoptotic and anti-apoptotic genes. Since Bcl-2, a potent anti-apoptotic regulatory protein, is believed to be a crucial mediator downstream of apoptotic signaling, we also assessed the effect of tryptanthrin on the expression of Bcl-2. Our study showed that Bcl-2 was highly expressed in K562 cells. The contents of Bcl-2 decreased while Bax (a potent pro-apoptotic regulatory protein) was elevated in K562 cells with increasing concentrations of tryptanthrin. The Bcl-2/Bax ratio significantly decreased in the presence of tryptanthrin.

Studies have revealed numerous proteins (such as COX and NOS) and cytokines (such as LT and IFN-γ) were targeted by tryptanthrin [11,22,23]. Because these proteins and cytokines were over-expressed during inflammation and carcinogenesis and mitochondrial damage plays a critical role in these processes, we investigated whether the apoptosis-inducing effect of tryptanthrin was through affecting mitochondrial function. The underlying events relevant to mitochondria were studied by measuring MMP. As expected, we observed a decline in MMP in the presence of tryptanthrin. The decline in MMP would lead to decreased ATP production and over-generation of oxidant, which is an early event in tryptanthrin-induced K562 cell apoptosis. Previous studies have also demonstrated that mitochondria are essential in the activation or amplification of the caspase cascade via the release of cyt-c from the mitochondrial intermembrane space [8]. Activation of the caspase cascade is considered as a critical sign that initiates the irreversible events in cell death. Cytosolic cyt-c is highly responsible for the activation of pro-caspase-3 [24]. Fei and colleagues extracted rutaecarpine (an agent having similar structural with tryptanthrin) from the Chinese medicinal plant Evodia rutaecarpa, and reported it can induce Hela cell apoptosis through cyt-c releasing [25]. In agreement with Fei’s report, our result here indicated that tryptanthrin-induced apoptosis is associated with mito cyt-c leakage, which is consistent with the activation of pro-caspase-3. Cytosolic cyt-c and active caspase-3 expressed in K562 cells in a low level. However, both cytosolic cyt-c and active caspase-3 (p20 and p17 subunits) contents were elevated in response to tryptanthrin administration. The contents of cytosolic cyt-c and activated caspase-3 subunits were in parallel with the tryptanthrin concentrations. The expressions of active caspase-3 subunits p20 and p17 were significantly abolished in the presence of a pan-caspase inhibitor ZVAD-FMK. Therefore, it is reasonable to believe that tryptanthrin might induce K562 cell apoptosis by the damage of mitochondrial membrane and the cyt-c-caspase-3 dependent mechanisms.

## 5. Conclusions

In summary, the present study highlights that tryptanthrin has proliferation-attenuating and apoptosis-inducing effects on the CML K562 cell line *in vitro*. Tryptanthrin triggers apoptosis associated mitochondrial breach. Disturbance of Bcl-2/Bax expression levels might participate in the apoptotic process and the underlying mechanisms might be cyt-c-caspase-3 dependent. Although we did not observe a better therapeutic effect of tryptanthrin than CTX in our study, tryptanthrin is free of adverse effect. Therefore, tryptanthrin might be a new candidate in the therapeutic strategy of CML.
